# Growth Stimulating Minimal Acetabuloplasty, Alongside Hip Open Reduction: A Simpler One-stop Operative Treatment for Developmental Dysplasia of the Hip

**DOI:** 10.1097/BPO.0000000000003259

**Published:** 2026-04-16

**Authors:** Alexander Aarvold, Nicholas Uren, Amanda Rhodes, Kirsten G. Elliott, Edward A. Lindisfarne, Belen Carsi, Nicholas M.P. Clarke

**Affiliations:** * Southampton Children’s Hospital, Southampton, UK; † University of Southampton, Southampton, UK; ‡ Royal Stoke University Hospital, Stoke-on-Trent, UK

**Keywords:** DDH, pelvic osteotomy, growth stimulation, open reduction, residual dysplasia

## Abstract

**Background:**

This paper describes a pelvic procedure that is technically simpler, quicker to perform, and equally effective as all existing pelvic osteotomies. It is used alongside hip open reduction for the treatment of the associated acetabular dysplasia. It is a minimal additional procedure that is intended to ignite the growth of the dysplastic acetabulum.

**Methods:**

A total of 167 hips (in 154 infants) treated with open reduction and growth-stimulating minimal acetabuloplasty, all with follow-up between 4 and 16 years postoperatively, are reported. The surgical technique is described. Sequential radiographs were analyzed through to final follow-up, which for 21% is to skeletal maturity. Patient demographics, preoperative and sequential postoperative indices, and outcomes are recorded.

**Results:**

Preoperative IHDI position was grade IV dislocation in 77% and grade III in 33%. Mean starting acetabular index (AI) was 41.3 degrees (range 30 to 54 degrees) and median age at operation was 13 months (range from 1 to 2.5years). At final follow-up, 98.2% of hips have a Severin 1 (excellent) or 2 (good) outcome, with the AI normalized. These are all IHDI grade 1 and have a mean center-edge-angle of 35.7 degrees. Only 6 of these (3.6%) have warranted a subsequent pelvic osteotomy for residual acetabular dysplasia.

**Conclusions:**

The growth-stimulating minimal acetabular procedure is technically straightforward. It is simpler than standard existing pelvic osteotomies, yet it is shown to be at least as effective. With up to 16 years follow-up, we can highly commend its routine use alongside hip open reduction in infants aged 1 to 2.5 years old.

**Level of Evidence:**

Level III—case-control study.

## BACKGROUND

This minimal acetabular procedure is a quick and easy technique that is designed to ignite growth of the dysplastic acetabulum when performed alongside standard hip open reduction in Developmental Dysplasia of the Hip (DDH).^1–3^ The existing options for managing acetabular dysplasia at the time of open reduction surgery for DDH are either nothing (with a consequential high chance of requiring further surgery a few years later in the form of formal pelvic osteotomy) or performing a large formal osteotomy (namely Salter or Pemberton^4–6^). Open reduction performed in isolation, without addressing the acetabular dysplasia, is standard practice in many institutions, including historically at our children’s hospital. There may be improved development of the acetabulum once a stable reduction has been achieved, but the further corrective surgery rate is high. The largest systematic review to date found this to be 56% following isolated open reduction, with the majority being for residual acetabular dysplasia (RAD). Multiple cohorts and registry data have identified the rate for RAD to be as high as 78%.^3,7,8^ Therefore, when open reduction is performed in isolation, there is a significant incidence of the infant being consigned to subsequent additional major interventional surgery in the form of pelvic osteotomies.^8^

There is therefore a rationale to address the acetabular dysplasia at the time of open reduction, through the standard anterior approach of Smith-Peterson.^9^ Surgeons who favor this regime currently tend to delay surgery beyond 18 months of age to allow increased size of pelvis and to make the Salter or Pemberton pelvic osteotomy technically more manageable.^6,10,11^ It is increasingly clear that the highest chance of producing the best possible result, without having to undergo further corrective surgery, is the formal open reduction through an anterior approach, combined with a pelvic procedure to address the acetabular dysplasia.^3,5,7,8^ However, both the Salter and Pemberton osteotomies are major interventions and technically quite complex. An open reduction is already a high-stakes and difficult operation, following which many surgeons may not be comfortable with the addition of a major pelvic osteotomy. Furthermore, the rotational aspect of the Salter uncovers the hip posteriorly, while the volume-reduction aspect of the Pemberton increases pressure on the femoral head.

This manuscript describes a growth-stimulating minimal pelvic procedure that is simple to perform and can take only a few minutes. It is much more easily performed alongside an open reduction than any existing pelvic osteotomy,^1^ without the associated disadvantages. This procedure has now been utilized at our institution for 16 years. It is through rigorous analysis of the longer-term outcomes, with many children now at skeletal maturity, that the merits of this procedure can be fully assessed and commended for uptake in routine clinical practice.

## METHODS

### Surgical Technique

**FIGURE 1:**
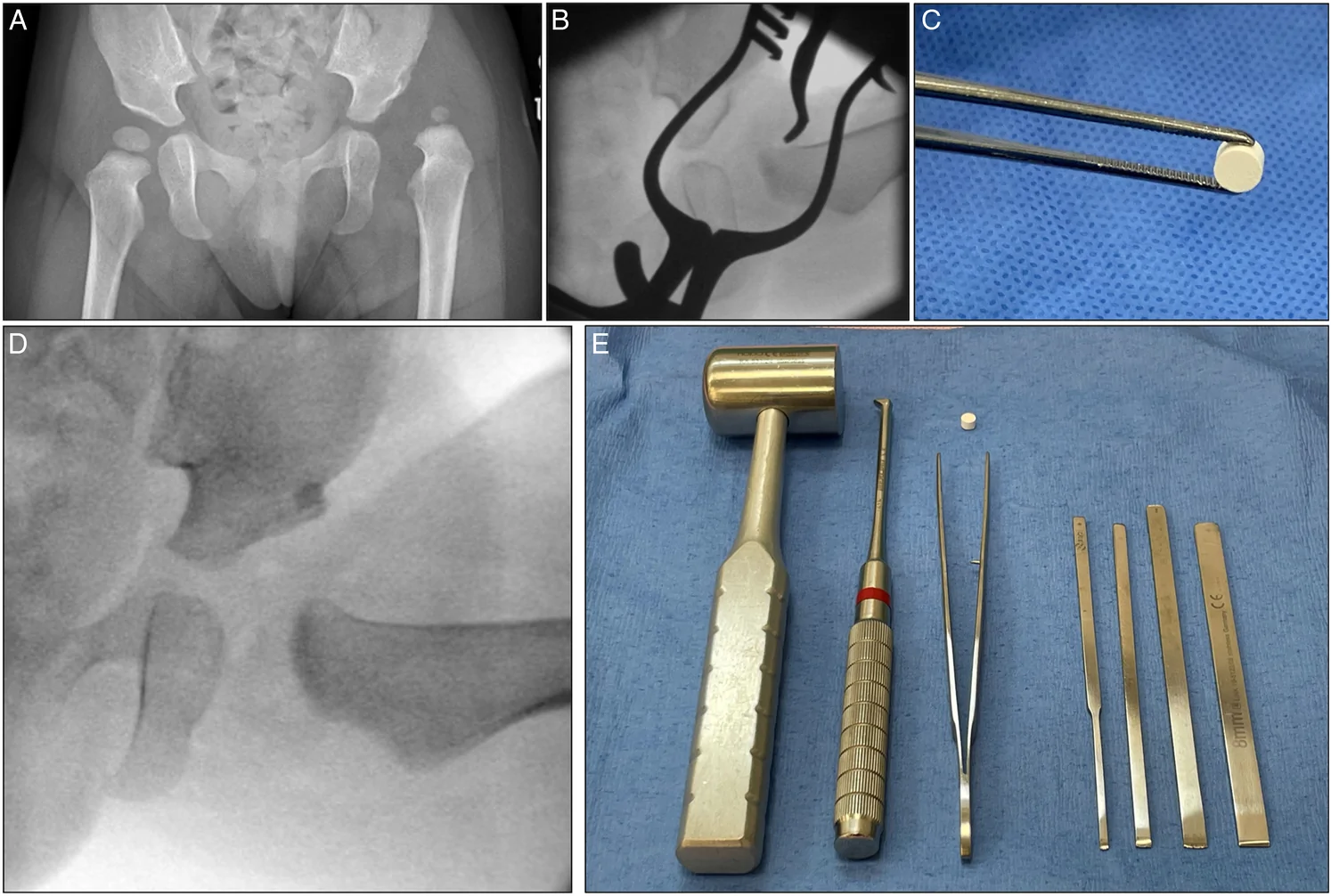
(A) Standard high-hip dislocation in a 1 year old; (B) hip restored concentrically in joint with formal open reduction; (C) the 3 mm CaSO_4_ pellet held in non-toothed forceps; (D) the pellet inserted in the upper outer aspect of the acetabulum; (E) the standard instruments needed to perform this small procedure— toffee hammer, small tamp, forceps, and straight osteotomes 2 to 8 mm.

First, the open reduction and capsulorrhaphy is meticulously performed as standard on a dislocated hip (Figs. 1A, B), through a Smith-Peterson approach that splits the apophysis. The unique aspect reported in this manuscript is the growth-stimulating minimal acetabular procedure that follows. This is achieved by placement of a small calcium sulfate pellet, measuring 3 mm in height (Fig. 1C) (OsteoSet, 4.8-mm diameter, Wright Medical UK Ltd., UK), just above the acetabulum in the outer table (Fig. 1D). The only instrumentation required is a mini osteotome set (widths range from 2 to 8 mm), which can be found on most standard orthopaedic trays or easily sourced (Fig. 1E). The technique has been described previously.^1^ The steps are reported here in detail alongside intraoperative photographs and fluoroscopy.

**FIGURE 2:**
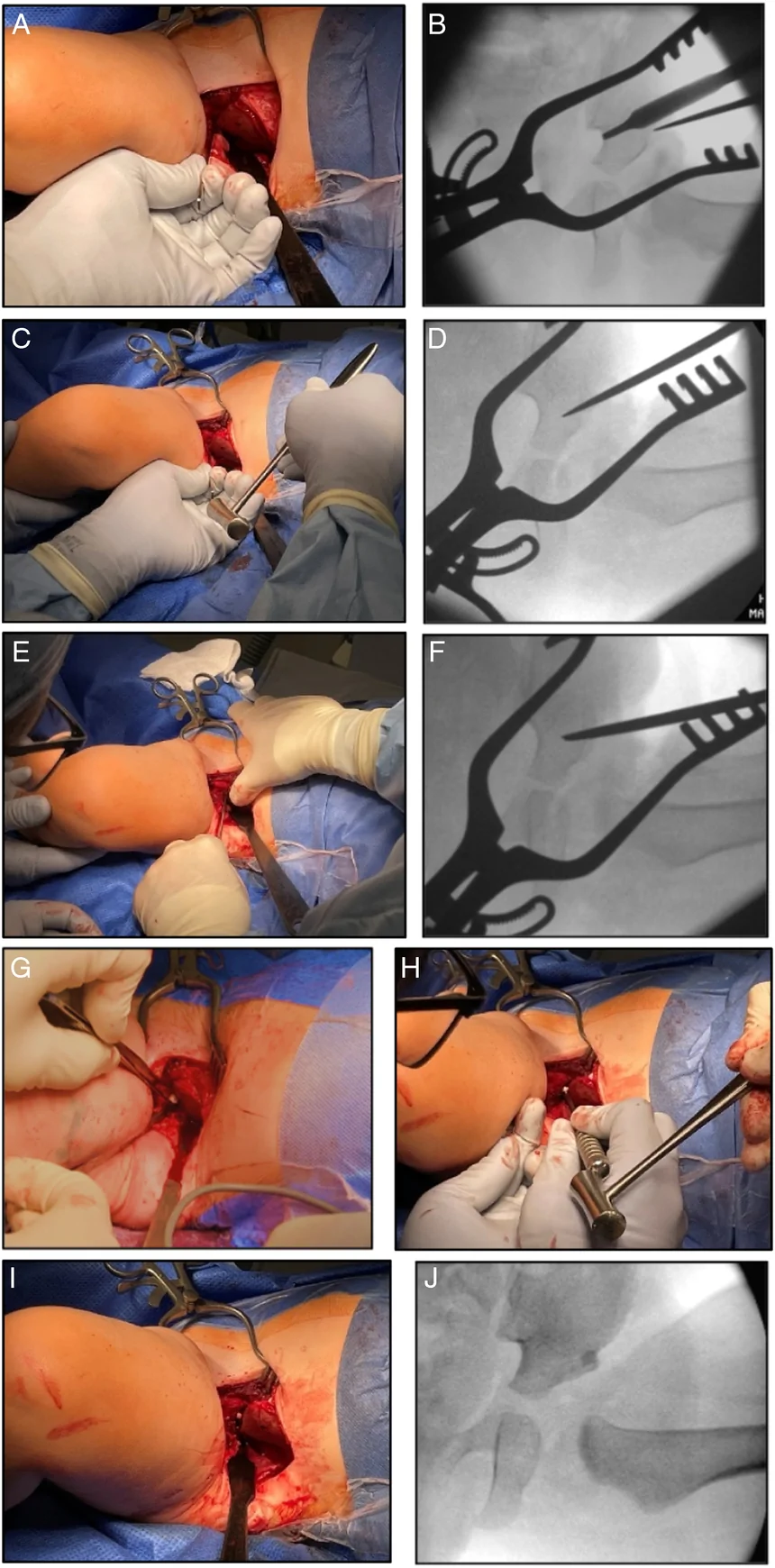
Intraoperative photographs and corresponding fluoroscopy images, demonstrating all steps in the minimal acetabular procedure. The steps are described in detail in the text under “Surgical technique.”

Figure 2 aligns intraoperative photographs with the corresponding fluoroscopy. Figure 2A demonstrates a left hip following open reduction and capsulorrhaphy—the patient’s head is to the right, and the flexed thigh is on the left side of the photograph. The 8-mm osteotome is placed in the mid-axial line just above the acetabulum (Figs. 2A, B). This is closer to the joint line than a Salter or Pemberton osteotomy. It is advanced into the outer table of the ilium using the toffee hammer, halfway across the acetabulum (Figs. 2C, D). This is usually around 1 cm in depth. A second osteotome (6 or 4 mm) is placed directly anterior to the first, and the 2-mm osteotome is used to break the anterior aspect of the iliac spine. The surgeon places a thumb on the iliac crest and gently levers the 2 osteotomes caudally to open a 3-mm gap in the outer table (Figs. 2E, F). The anterior osteotome is removed while the 8 mm osteotome holds the gap open, and the pellet is placed into the anterior gap (Fig. 2G). This is tapped in using the tamp and toffee hammer (Fig. 2H). The 8-mm osteotome is removed, leaving the pellet held in position (Fig. 2I). The acetabulum is not fully corrected (Fig. 2J), as it would be at the end point of a Salter or Pemberton osteotomy, but the technique is designed to stimulate the growth of the anterior and lateral part of the dysplastic acetabulum. Postoperatively, patients are in a spica cast for 6 weeks, an abduction cast for 6 weeks, and an abduction night splint for 6 weeks.

### Cohort Analysis

This technique has been performed at our institution since 2009, implemented as a result of the high rate of RAD after open reduction alone.^3^ Five surgeons at our center have been performing this, with our standard practice for DDH open reductions being double consultant operating. The indications are any infant who is undergoing a formal open reduction of their dislocated hip, up to the age of 2.5 years (ie, 30 mo). The acetabulum does become less pliable with age; thus, when an infant has a late-detected hip dislocation after the age of 2.5 years, the practice at our institution is to perform a formal, larger traditional pelvic osteotomy (ie, Pemberton or Salter) rather than the growth-igniting minimal procedure that is the subject of this analysis.

All DDH post-surgical patients remain under continued follow-up through to skeletal maturity, as standard practice at our institution, many of whom are part of the Global Hip Dysplasia Registry (GHDR).^12^ Review of all cases was approved by the University of Southampton Ethics Research & Governance Online (ERGO) board, Ref 60802.

Patient demographics were noted, including baseline age at diagnosis, age at surgery, hip laterality, and pathway to surgery (ie, failed Pavlik harness, failed closed reduction, or late detection). Teratological hip dislocations (ie, those in patients with neuromuscular syndromes) were excluded to focus on true DDH.

All children who have reached a minimum of 4 years of follow-up are included in this study, to report only on the medium to longer-term outcomes. Less than 4 years of follow-up is insufficient time to capture key outcomes such as type 2 avascular necrosis and the re-operation rate for further corrective surgery. Before the age of 5 years, it is not feasible to measure the center-edge angle and Severin classification, due to the partly ossified proximal femur and acetabulum, which are key radiographic indicators following open reduction surgery. Ultimate follow-up would be function into mid-adulthood, though there are very few studies that have managed this.^13^ Radiographic hip morphology is a good surrogate for functional outcomes.^14^ At up to 15 years follow-up in this study, many children have reached skeletal maturity (defined by the closure of all physes) and have been discharged from the children’s orthopaedic clinic.

Every radiograph for every patient was reviewed on the hospital Picture Archiving and Communication System (PACS). All families who have moved away had their follow-up images imported for review. Preoperative measurements were the acetabular index (AI) and International Hip Dysplasia Institute (IHDI) classification.^15^ Postoperatively, AI was recorded for every sequential radiograph, enabling sequential growth to be plotted. Center-edge angle of Wiberg (CEA) was measured on the most recent radiograph for each patient, alongside IHDI classification and Severin grading.^16,17^ In all patients with unilateral DDH, the normal contralateral hip parameters were used as normal comparators of AI. All radiographic measurements were performed by at least 2 independent reviewers, with any discrepancy being returned for consensus decision.

## RESULTS

### Cohort Characteristics and Follow-up Duration

There are 167 hips with DDH in 154 infants who have had the growth-stimulating minimal acetabular procedure alongside their standard hip open reduction, and who have reached at least 4 years of follow-up post-surgery. All hips were frankly dislocated preoperatively, with 129 of 167 (77%) being IHDI grade IV (high dislocations). The remaining 38 hips were IHDI grade III.

There are 133 females and 21 males, in keeping with the usual sex discrepancy of DDH. Median age at operation was 13 months, with a range from 11 to 30 months, that is, from 1 to 2.5 years old. All have a minimum of 4 years postoperative follow-up, even if there were some missed appointments in the interim, particularly during the COVID pandemic. There are 32 patients who have reached skeletal maturity and have been discharged. Their ages ranged from 13 to 17 years old at discharge. Thus, the duration of follow-up ranges from 4 to 16 years following surgery (5 to 17 years of age). Mean follow-up is to 10.4 years of age (SD 3.4) and 9.2 years (SD 3.5) postoperatively.

There were 69 of 154 babies (45% of the cohort) who failed Pavlik harness treatment (due to persistent fixed hip dislocations or recurrent femoral nerve palsy, or a combination of both). The remainder (85 infants) were late-detected hip dislocations (including one bilateral), with an age range from 4 to 27 months at late diagnosis. There were 12 hips that had had a failed closed reduction before their open reduction minimal acetabuloplasty surgery.

There were 41 contralateral hips that also had DDH, leaving 126 normal contralateral hips for whom serial acetabular index (AI) was measured as a normal baseline. The 41 contralateral hips with a DDH diagnosis had treatment that included simultaneous closed reductions or sequential hip open reduction with growth-stimulating minimal acetabular procedure. The latter group was 13 children, all of whose bilateral hips are the subject of this analysis.

### Objective Radiographic Measurements

**FIGURE 3:**
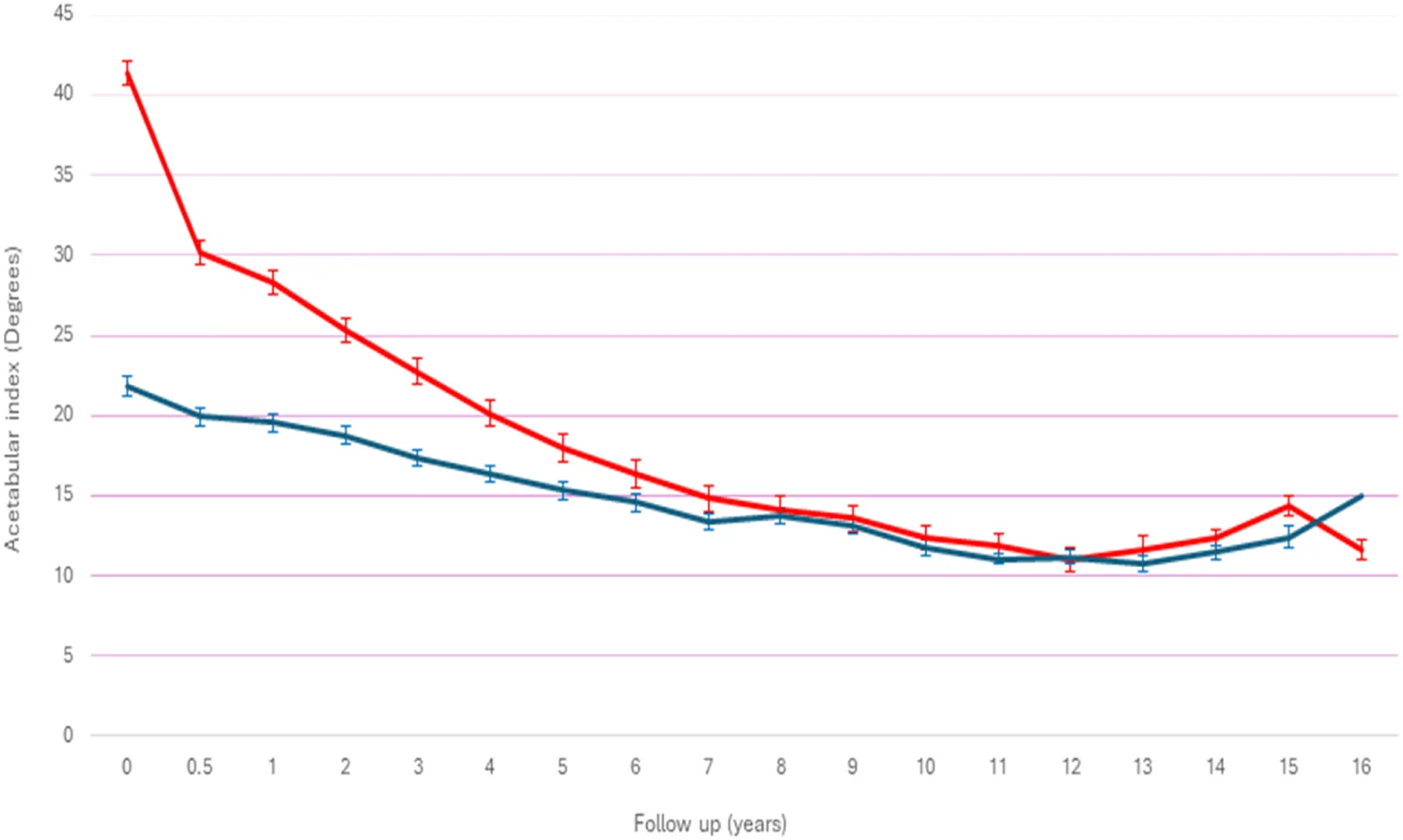
Sequential acetabular indices (AIs) of hips treated with open reduction and growth-stimulating minimal acetabuloplasty (mean and standard deviations). The blue line represents the contralateral normal hips (n=126). The red line is the DDH hips (n=158), showing sequential acetabular development to the normal range.

Median preoperative AI of the dislocated hips was 41.3 degrees (range 30 to 54 degrees), compared with 21.8 degrees (range 10 to 32 degrees) for the normal contralateral hips. The mean sequential AIs following open reduction with growth-stimulating minimal acetabuloplasty are displayed in Figure 3 as the red line, with standard deviation bars. The bottom line shows the AI of the contralateral normal hips (blue), for comparison of normality. From a starting AI of 41.3 degrees, ultimate normalization of the dysplastic acetabulae is demonstrated. The dysplasia is partially corrected with the minimal opening wedge and placement of the CaSO_4_ pellet. At the first postoperative XR, the AI averaged 30.2 degrees, so it was still clearly undercorrected acetabular morphology. However, by 3 years postoperatively, the AI of the operated hips is, on average, 22.7 degrees. With each sequential year, the measured AI of the operated hip becomes closer to the normal contralateral hip. At 4 years following surgery, the difference is within 4 degrees of the contralateral normal hips. By 7 years postoperatively, the SD bars overlap, and AI of the operated hips are statistically indistinct from the normal hips (*P*=0.07, unpaired *t* test).

**FIGURE 4:**
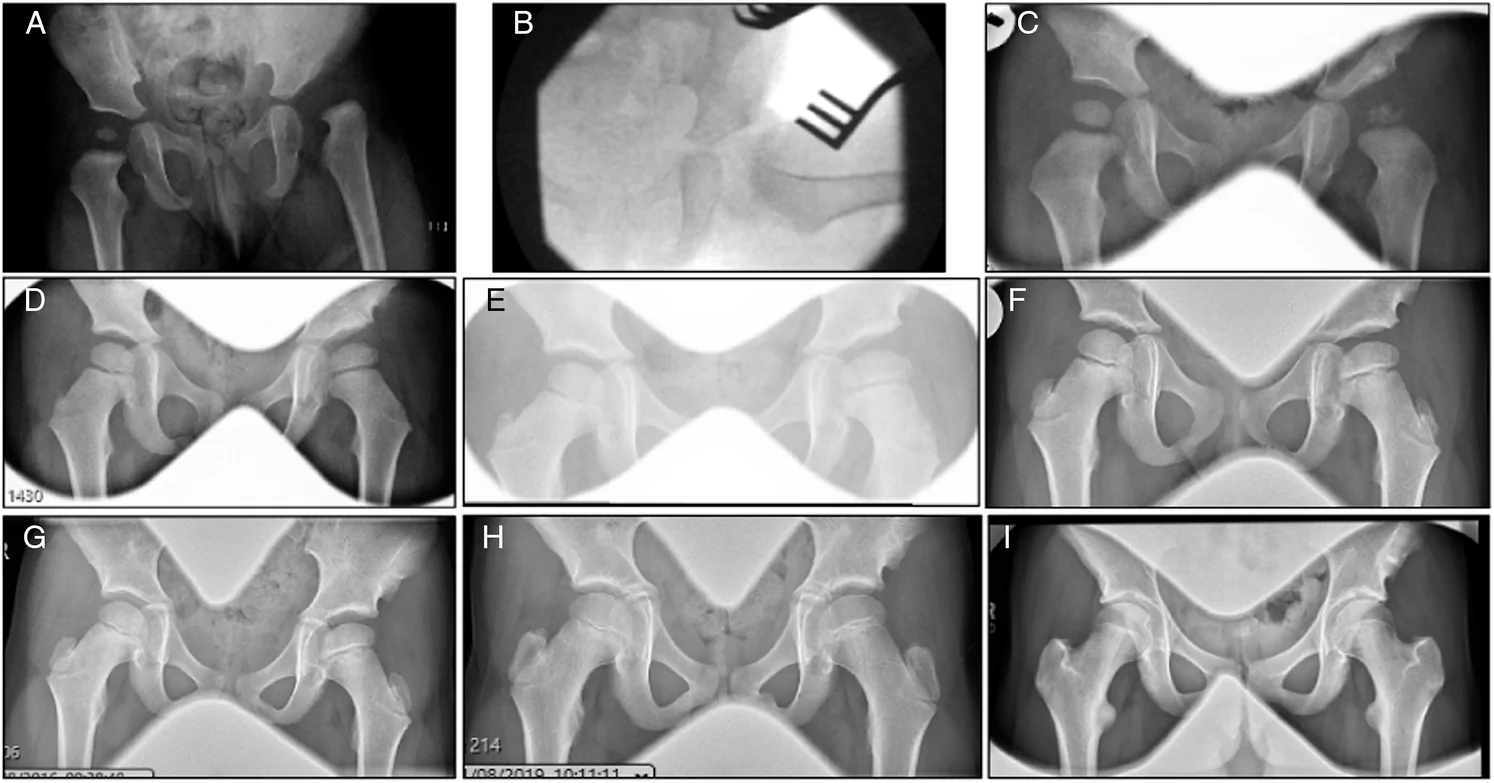
Serial radiographs following left hip open reduction and growth-stimulating minimal acetabuloplasty, in a girl who is now 14 years old and at skeletal maturity. (A) Preoperative showing IHDI 3 hip with an AI of 41 degrees, compared with 23 degrees on the normal right hip. (B) Intraoperative fluoroscopy at 11 months of age, showing the pellet in the upper outer edge of the acetabulum. (C–H) Progressive development of the hip and the AI at 1, 2, 3, 4, 5, and 8 years following surgery. (I) Thirteen years postoperative: AI 5 degrees and symmetrical; CEA 43 degrees; Severin 1; asymptomatic with normal, symmetrical clinical examination.

Figure 4 is an example of the serial growth that is observed in comparison to the normal contralateral hip. This child has now reached skeletal maturity and has been discharged from our children’s hospital clinic. She has no symptoms from her hip and continues to take part in all normal sports.

**FIGURE 5:**
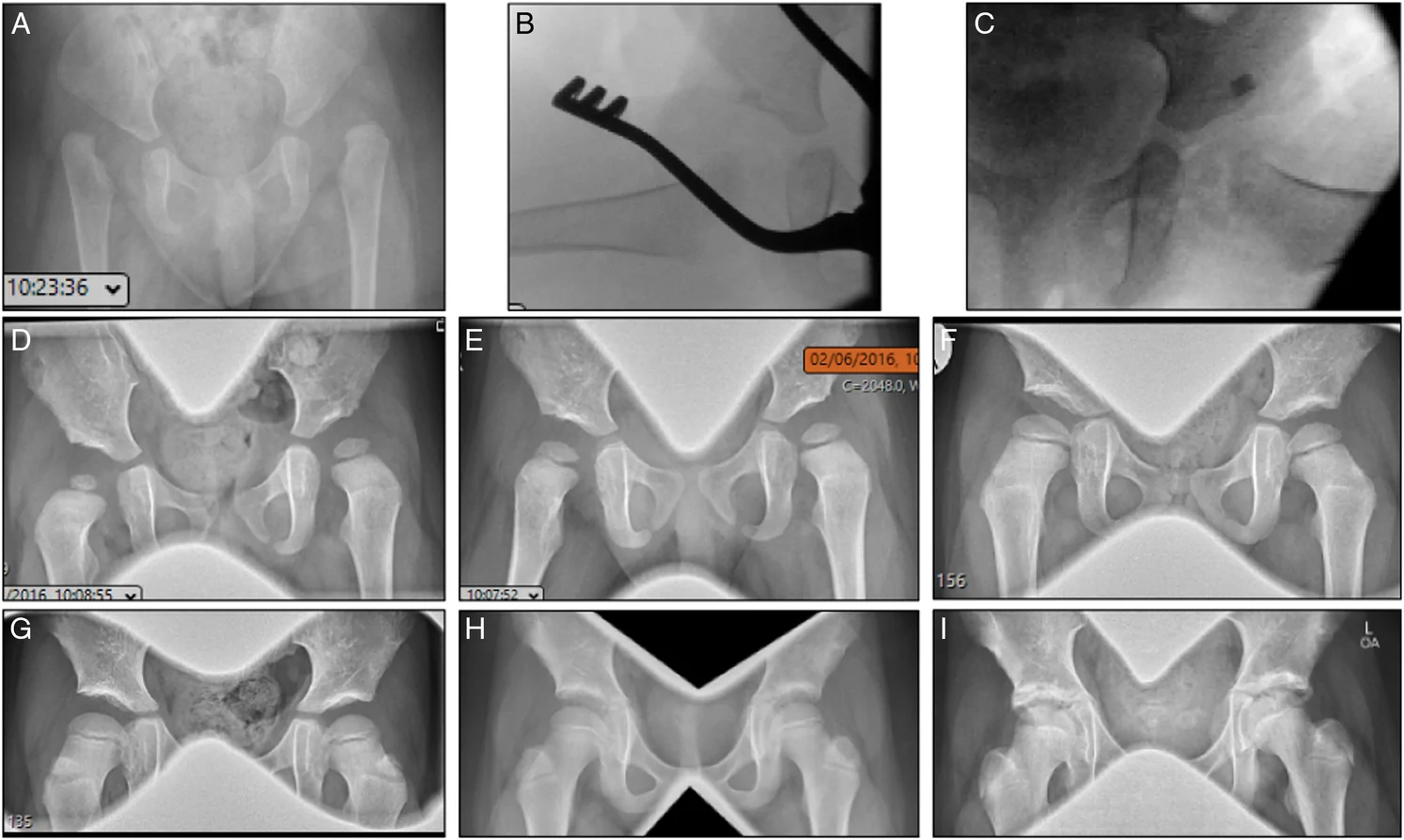
Serial radiographs following bilateral sequential hip open reduction and growth-stimulating minimal acetabuloplasty, performed at 13 and 17 months of age. (A) Preoperative showing IHDI 4 hips with AIs of 38 degrees (right) and 36 degrees (left). (B, C) Intraoperative fluoroscopy, showing the pellet in the upper outer edge of the acetabulae. The left side was operated on first at 13 months of age, followed by the right side 5 months later. (D, E) One year following left and right hip surgery, respectively. (F, G, H) Progressive development of both hips and the AI at 2, 3, and 6 years following surgery. (I) Ten years postoperatively: right AI 3 degrees/CEA 55 degrees, left AI 6 degrees/CEA 52 degrees, both hips essentially symmetrical and Severin 1, asymptomatic.

Figure 5 is an example of an infant with bilateral hip dislocations who had sequential hip open reduction and growth-stimulating minimal acetabuloplasty. The left side was operated on first, and the right side 5 months later. The key observation is the slight early correction at 6 to 12 months following the small lateral opening wedge and pellet placement, but the acetabulum would be considered still dysplastic. The ongoing acetabular growth over the subsequent few years is demonstrated in sequential radiographs. This child is 11 years old at the latest follow-up, playing all sports without any limitation or any symptoms from her hips.

**FIGURE 6:**
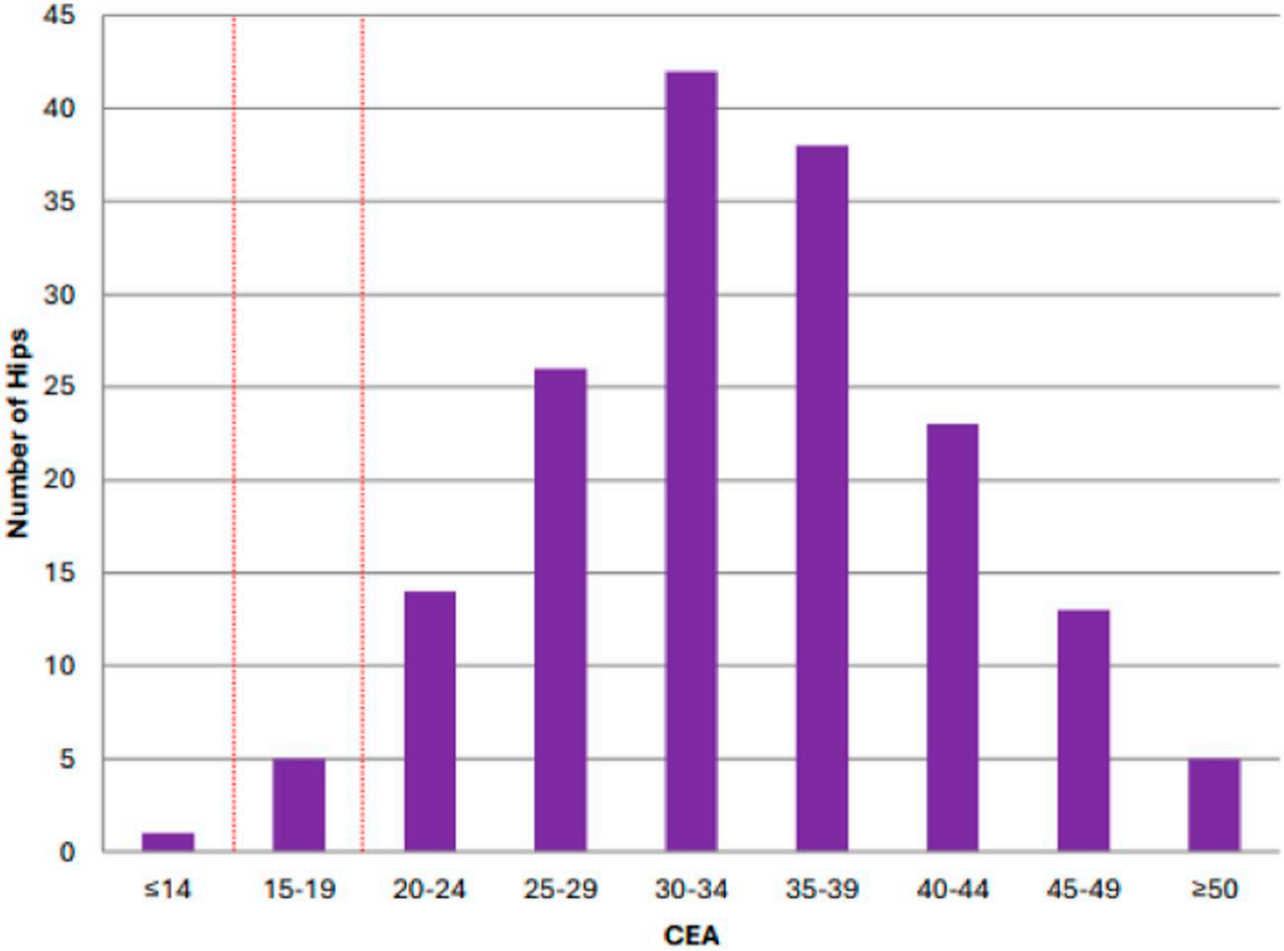
Center-edge angle (CEA) at final follow-up of all 167 hips treated with open reduction and growth-stimulating minimal acetabuloplasty. The lower limit of the normal CEA range is marked as the 2 red lines, namely 15 degrees for children aged 6 to 13 and 20 degrees for children aged ≥14 years.

Of 167 dislocated hips treated by open reduction and growth-stimulating minimal acetabuloplasty, 98.2% have a Severin 1 (excellent) or 2 (good) outcome at a mean age of 10 years. The mean CEA of this cohort at the most recent follow-up is 35.3 degrees (range 14 to 55 degrees), illustrated in Figure 6. While all hips were IHDI grade 3 or 4 preoperatively, 98.2% were IHDI grade 1 at the most recent follow-up, with 3 hips being IHDI grade 2.

There are 92/167 hips (55.1%) that are Severin 1 (excellent) at final follow-up, 72/167 (43.1%) that are Severin 2 (good), and there are 3 hips (1.8%) that have a Severin outcome of 4a (poor), namely subluxation and deficient acetabular coverage. The CEA of these 3 poor outcome hips are 14, 18, and 20 degrees at 14, 7, and 10 years old, respectively. One has had further corrective surgery [femoral varus osteotomy (FVO)], and all remain under ongoing review.

### Residual Acetabular Dysplasia, Further Corrective Surgery, and Complications

There are 6 of 167 hips (3.6%) that have come to a later pelvic osteotomy for residual acetabular dysplasia (RAD). All had a Pemberton pelvic osteotomy, with 3 having concomitant FVO. These 6 patients had their further operations at 3, 5, 5, 8, 10, and 11 years following the index surgery. Their age at primary operation was 11, 11, 17, 18, 24, and 26 months, representing a spread across the age cohort. All are Severin 2 outcomes at the most recent follow-up, due to the varus alignment of the proximal femur or surgical change in the acetabulum. It is worth noting that the formal pelvic osteotomy in all cases was uncomplicated, as the operation was being performed on essentially unspoiled tissue/iliac bone. This is in comparison to the senior authors’ experience of doing formal pelvic osteotomies after previous Pemberton/Salter osteotomies have been performed.

**FIGURE 7:**
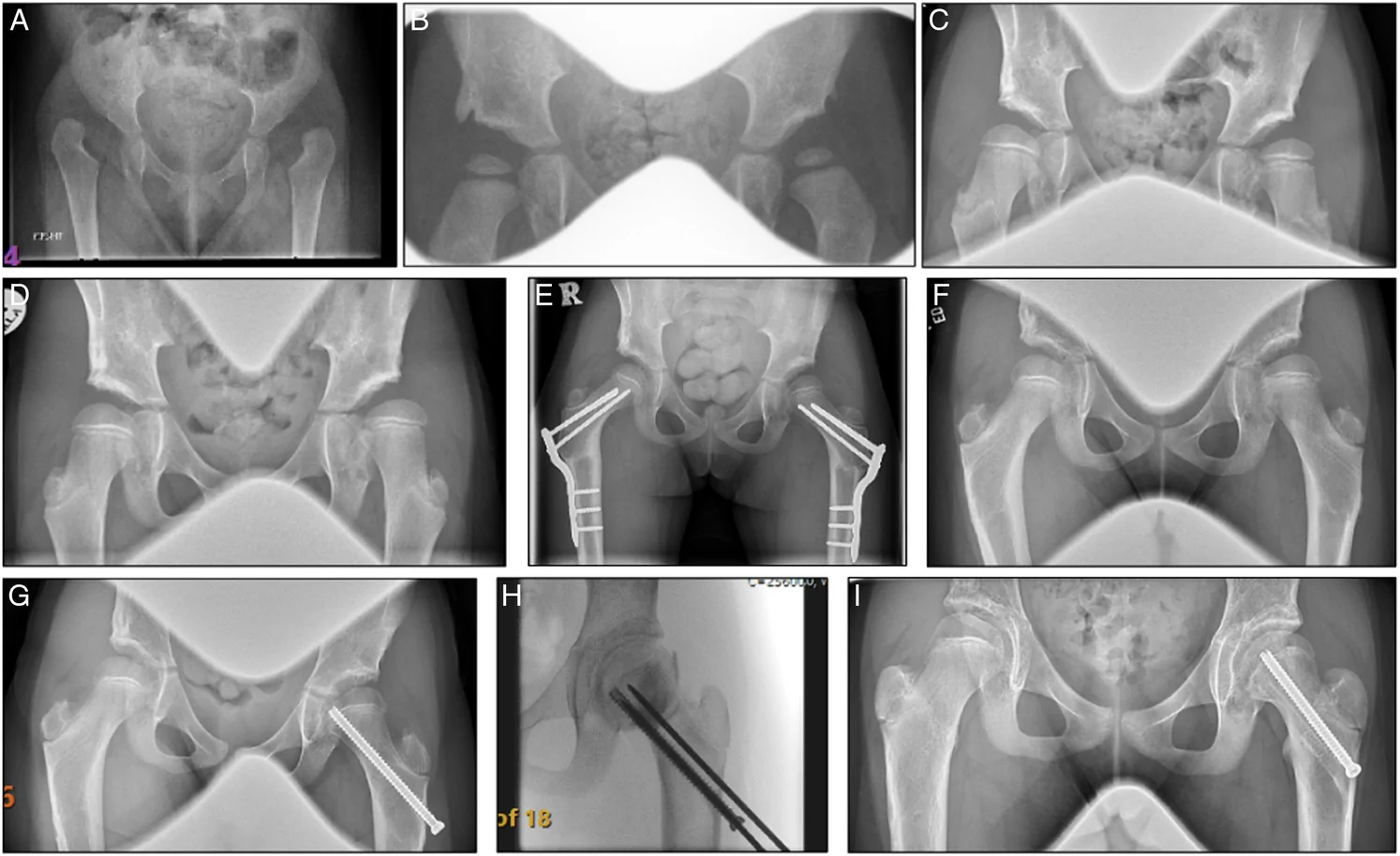
Serial radiographs following bilateral sequential hip open reduction and growth-stimulating minimal acetabuloplasty, at 11 and 16 months of age, plus subsequent further corrective surgery (FCS) for lateral growth arrest. (A) Preoperative showing IHDI 4 hips with AIs of 41 degrees (right) and 40 degrees (left). (B, C, D) At 1, 4, and 6 years following surgery, showing progressive lateral growth arrest and an increase in neck-shaft angle (NSA), left worse than right. Despite this hip displacement, the acetabulae have continued to mature. (E) Three months following bilateral FVO, demonstrating restoration of concentric position of both hips. (F) Recurrence of increased NSA on the left side at 2 years following FVOs. (G) A medial femoral head screw was placed to halt further progression at 9 years following index surgery. (H) An exchange screw was placed after the first screw had grown off the physis at 11 years postoperatively. (I) Twelve years postoperatively: right AI 9 degrees/CEA 36 degrees; left AI 11 degrees/CEA 33 degrees; both hips Severin 2. At 13 years old, the patient is asymptomatic and participating in all sports without limitation.

A total of 25 children (15.0%) have undergone further corrective surgery in this cohort. Two hips re-dislocated (1.2%), treated with prompt revision open reduction. Six hips had POs for RAD, as described above. The remainder have had surgery for later lateral growth arrest, in keeping with the slow development of a type 2 AVN picture. These operations have been a mixture of medial femoral head screw epiphysiodesis or FVOs, or sequentially both. These operations for type 2 AVN occurred anywhere from 3 to 11 years after the index procedure, with a sequence illustrated in Figure 7.

Three other complications occurred: 1 distal femur buckle fracture a few months after removal of spica (managed in a single-leg spica), 1 proximal femur osteomyelitis, and 1 superficial wound infection (both treated with antibiotics).

## DISCUSSION

This growth-stimulating minimal procedure is highly effective in the treatment of acetabular dysplasia. It does not fit into any existing categories of pelvic osteotomy. It is not rotational (like a Salter, Triple, or PAO) nor is it volume reducing (like a Pemberton, Dega, or San Diego) nor is it salvage (like a Chiari or a Shelf). This acetabular procedure is a theorized growth-stimulating minimal osteotomy, in a category of its own. Though the volume of the acetabulum is partially reduced by the opening of the lateral aspect of the acetabular lip to insert the pellet, the early mean postoperative AI demonstrates an uncorrected acetabulum (mean 30.2 degrees at first follow-up, down from a mean of 41.3 degrees preoperatively). Its effectiveness in stimulating subsequent growth has been clearly demonstrated in this manuscript—by the number of cases in which it has been performed, by the extended duration of follow-up, by the sequential AI improvement to normal range seen in Figure 3, and by the excellent longer-term outcomes to skeletal maturity. The early promise of this procedure has been borne out. That is why we can highly recommend that this procedure should be considered alongside DDH open reduction, in children aged 1 to 2.5 years old, in settings where pelvic osteotomies are not performed routinely.

One could argue that the acetabular development seen following this procedure is just the natural progression after the hip has been concentrically reduced. Any surgeon who manages DDH can share examples of good acetabular development after early restoration of the hip into a concentric position by closed or open reduction, despite nothing being done to the acetabulum, as can the authors of this manuscript. However, even if a closed or open reduction achieves concentric hip, and achieves it early, the rate of RAD remains high. Multiple observational cohorts demonstrate that RAD is a significant challenge following isolated open reduction, including a recent prospective multicenter trial providing robust level II evidence, highlighting that RAD is as high as 78% in isolated open reductions.^3,7,8^ With an incidence of 5.4% of RAD (demonstrated by 6 out of 167 cases in this cohort requiring pelvic osteotomy, and 3 cases of Severin 4a), one can be confident that this minimal acetabular procedure is having a major effect.

Every hip in this series was irreducible by closed means, determined either by a previous failed closed reduction or an arthrogram at the time of surgery. The majority of hips were high dislocations (77% were IHDI grade IV) and with high starting AIs (median 41.3 degrees). Thus, the hips in this series are as severely affected as any published series to our knowledge. Yet the results are excellent, with advanced acetabular development after only minimal acetabular intervention.

The earliest results of this procedure were published in 2014, with a mean follow-up of 4 years.^1^ The trajectory of those early results exactly mirrors the results reported here of larger numbers of patients and with longer follow-up. This is a good indicator of the expected onward good trajectory of those hips with several years of growth and follow-up still remaining. Of the 2 hips that re-dislocated, a formal pelvic osteotomy was not needed to stabilize either hip at revision open reduction. Both these hips remained enlocated, with continued acetabular development on track with the sequential AIs illustrated in Figure 3. Where type 2 AVN has occurred, creating a lateralized hip position and loss of concentricity, the acetabular development usually remains unaffected and on track to an excellent final shape. This is suggestive of independent acetabular growth stimulation, not reliant on a concentrically reduced hip, which is illustrated in Figure 7.

The main limitation of this study is that it is not a randomized controlled trial (RCT) but a single-center observational cohort. The evidence base would be strengthened by a prospective, ideally clustered, RCT. However, with the outcomes demonstrated in this manuscript compared to isolated open reduction,^3,5,8^ it would not be appropriate at our center to regress and leave some babies with their acetabulum untreated at the time of open reduction. Since 2017, all families have been offered inclusion in the Global Hip Dysplasia Registry, enabling prospective data collection.

True rates of further corrective surgery may be underestimated, as not all patients in this cohort have reached skeletal maturity. Despite a minimum follow-up of 4 years, there may be a few of the younger children who come to secondary intervention in their remaining growing years. Further analysis is scheduled when all these patients have reached skeletal maturity.

There are 9 hips (5%) omitted from the data in Figure 3. These are the 3 that have a poor outcome (Severin 4a) plus the 6 that have had a subsequent PO for RAD. Their omission narrows the error bars but is important as this enables Figure 3 to be the standard against which expected acetabular hip development can be matched, following the growth-stimulating minimal acetabuloplasty. Should a hip be deviating from the trajectory illustrated in Figure 3, further pelvic surgery should be considered.

A further limitation of this concept is that the mechanism of growth stimulation is not entirely clear. Graft composition is unlikely to be the key factor; indeed, other centers in which this technique has been adopted take a similarly sized pellet from the iliac crest. It is possible that just the mini osteotome insertion is the critical step (in a similar way to growth stimulation after microfracture). Perhaps the partial correction creates a more favorable environment for continued acetabular remodeling around a concentric hip. Whatever the exact biological mechanism, the steps described in this manuscript are robust, repeatable, and highly effective in nearly eliminating RAD after open reduction. The strengths of the study, despite being single-center, lie in the cohort size, duration of follow-up (to 16 years following surgery and with 21% of children at skeletal maturity), and data completion. The numbers (167 hips and 154 patients) reported are higher than most series of DDH surgery outcomes. Thus, it is with considerable confidence that we can recommend this procedure to be readily adopted. Surgeons who are confident in performing a Salter or Pemberton osteotomy alongside an open reduction may have no reason to change practice. However, for any surgeon who would perform an isolated open reduction without addressing the pelvis, for whatever reason, the minimal addition of this quick procedure should be an easy and comfortable thing to do. It can be performed more easily at the end of a hip open reduction than a Salter or Pemberton. There is no extra exposure required beyond what one would normally do for the standard open reduction. Furthermore, this can be done from 12 months of age, as the pelvis does not need to be any particular size for this procedure to be performed. This should avoid the need for additional later pelvic osteotomies in a major proportion of their patients.

The descriptive term used throughout this manuscript is of a “growth-stimulating minimal acetabuloplasty,” yet for which there is no obvious mnemonic. Our unit has been colloquially calling this operation an “open reduction-capsulorrhaphy-acetabuloplasty” or “ORCA.” However, the term “acetabuloplasty” is fairly generic and is frequently used when describing existing bigger acetabular procedures. It does not adequately convey the uniqueness of this growth-stimulating minimal procedure. “Pelletoplasty” has been coined to refer to the CaSO_4_ pellet placed into the outer lip, also “feathering” or “tickling” of the acetabulum to describe the minimal nature of the procedure.

In summary, after a surgeon has completed an open reduction of a dislocated hip, this growth-stimulating minimal acetabular procedure is technically straightforward and quick to perform. It is relevant up to the age of 2.5 years, it is less invasive than the formal pelvic osteotomies of Salter or Pemberton, and appears at least as effective. Now, with up to 16 years of follow-up, we can confidently recommend this technique to be performed routinely alongside hip open reduction.
